# Dental Items

**Published:** 1871-05

**Authors:** 


### EDITORIAL DEPARTMENT.

*Dental Items.*—We have received from Dr. Jas. F. Thompson of Fredericksburg, Va., a beautiful specimen of "Calcified Pulp," as a contribution to the museum of the Baltimore College of Dental Surgery. It is one of the best specimens we have met with, and the following is the history of the case as related by Dr. Thompson:

"It is a second left superior molar, from the mouth of a lady, who came to me suffering intensely with what I supposed to be periodontitis. I gave the tooth a thorough examination, and found, to my surprise, no appearance of caries except very superficial, and the color normal. I debated sometime with myself as to whether I should extract the tooth, being a conservative practitioner. I was somewhat afraid that such an operation would reflect upon my skill, but concluded that where there was was so much inflammation of the lining membrane, the nerve must be involved, and with a demand on the part of the patient to extract the tooth, I did so and found, as the cause of the trouble, a calcified pulp. Had I suspected anything of this matter, I believe I could have saved the tooth, but such a diagnosis, unfortunately, in this case, owing to the soundness of the tooth was difficult to arrive at."

The following cases of calcified pulp are narrated in a recent number of the *London Lancet:*

"Mr. White, at the recent annual meeting of the Odontological Society, made an important contribution to an understanding of such cases. A boy eight years old was brought to him on account of continued pain in a lower temporary canine tooth. The tooth being quite sound and firm, he advised that the gum be well rubbed with spirits of camphor as a counter-irritant, and that the boy be brought back in a week. But before this time had elapsed the boy was brought back with a request that the tooth might be at once extracted, with a view to the relief of the incessant pain. This was done, and the cause of the pain looked for in the pulp cavity of the tooth by Mr. White, who at a former time had found intolerable pain caused in a young lady of seventeen, in whom all the teeth on the affected side were apparently sound. She referred the pain to an inferior front bicuspid. This was eventually extracted, at the earnest solicitation of the patient, and on being opened the cause of pain was manifested in a globular mass of osseous deposit in the upper part of the pulp. It consisted of nodular dentine pressing the sensitive pulp against the walls of the cavity. In the case of the little boy, Mr. White's scientific curiosity was rewarded by finding in the pulp an osseous deposit unlike either secondary or nodular dentine. It was more like very coarse cementum; but on closer examination it was found more nearly allied to osseous tissue existing in the cancelli of bone. It filled very nearly the whole of the pulp cavity at the junction of its middle and lower thirds, and was pressing the fascicula of nerve fibres out of their course. There can be no doubt that this was the cause of the pain. Two months had elapsed, and Mr. White had heard nothing more of his little patient."

"The *British Medical Journal* narrates the following case of "swallowing of artificial teeth," one of which proved fatal:

Mr. Henry Smith showed to the Medical Society of London a gold tooth-plate and teeth that had been swallowed by a gentleman, a patient of Dr. Hamilton, of Mitcham. Mr. Smith saw him six hours after the accident, and was then able to touch the plate with a pair of long forceps; but attempts at extraction were unavailing. Mr. Smith therefore pushed the plate down into the stomach. The patient felt relief; and nine days afterwards passed the plate by the bowel, with but little pain. Mr. Smith also showed a similar plate in vulcanite, given him by Dr. Johnson, that had been swallowed, and passed through the patient in safety. Dr. King narrated the case of a man in the Edinburgh Infirmary who had swallowed his false teeth. Mr. Syme not being able to pull the teeth up, at once pushed them down. A few days afterwards Dr. King was sent for to see the man, and found him dead. The angular hooks on the tooth-plate had torn the œsophagus and perforated the aorta. In the stomach was a complete cast of its cavity in blood-clot. Mr. Carter had seen a case where a brooch was swallowed. The patient was made to eat a large quantity of bread, and then an emetic was given, when the bread and brooch all returned together. Dr. Morrell Mackenzie was in the habit of using in such cases an instrument known on the continent as the *ramoneur*. It was passed down, and, as it was withdrawn, a sort of brush, expanding, caught the foreign body, and so removed it.

The following interesting case is from the *London Lancet:*

Emma N——, aged twenty-five, whilst in bed on Christmas-eve, dreamed that she was swallowing something hard, and on waking discovered that three artificial teeth fixed to a plate of silver and platinum had disappeared from her mouth. She experienced no difficulty at all in breathing. She could not swallow any solid food, the attempt to do so producing a feeling of sickness; but beef-tea, milk, and broth she found no difficulty in swallowing.

The patient was brought into the operating theatre on the 11th inst. Mr. Walton passed a pair of long forceps without meeting with any obstruction. He then passed an œsophagus bougie for the purpose of exploring. The instrument entered the stomach, but grated against something in its passsge down the tube. The bougie on being withdrawn showed scratches caused by some foreign body. Judging from the upper—i. e.,the last—part of the scratch, the object must have been in the œsophagus, at a point opposite the insertion into the sternum of the third costal cartilages. The scratch itself measured 9 in. in length, and there was about the same distance from the upper part of the scratch to the point of the bougie which touched the patient's lips during the exploration. Mr. Walton then passed a hooked probang, and caught hold of something that appeared to have a sharp edge. He then withdrew the object slowly and carefully to avoid lacerating the œsophagus. When, however, it had been brought to the upper part of the pharynx, the patient made a sudden gulp, and swallowed it again. Mr. Walton then seized a pair of long, curved forceps, and introducing them into the œsophagus, grasped the artificial teeth, and recovered both them and the plate. No loss of blood ensued, and there was only a slight reddish tint in the mucus which the patient spat out.

Although the metallic plate had been out of order for a long time previous to the accident, the patient had never taken the precaution of removing it at night. It held three teeth on one half of its irregular convex edge. Its entire length was 1⅝ in., and the greatest breath 1 inch.

As the operation produced pain, and there must have been more or less tearing of the œsophageal surface, Mr. Walton deemed it advisable that the patient should, for a time, take no food by the mouth. For the first four days she therefore received enemata of six ounces of beef-tea; for three days more these were increased to eight ounces, and administered every five hours. They were all retained. She then began to take liquid food, and shortly afterwards was placed upon full diet. She left the hospital quite well, and without having shown any appreciable loss of strength or flesh.

At a recent meeting of the "Miss. Valley Dental Association" Dr. Watt, in a discussion on the "treatment of the soft tissues of the mouth," remarked :

"One condition of mouths we have frequently to observe and not attribute to other causes, is the "nursing sore mouth of women." Generally an impoverished condition of the blood accompanies this disease. The treatment of these cases is so affected by the constitutional conditions of the patient, that we are frequently too restricted in our treatment, *i. e.* we treat too topically. Sometimes these cases are treated with nitrate of silver or carbolic acid, every day, or every other day without benefit. The treatment is wrong. The blood must be perfect. Physicians used to say to the mother with this sore mouth' "you must wean your child and stop this drain, as the impoverished condition of your blood will not bear it." Thinks this is in a large majority of cases unnecessary. The zymotic diseases are of this character, produced as they are by foreign substances in the blood. having less vitality than the blood. This virus must be got rid of, and if we can so adjust our remedies that they will kill this poison, and not affect the healthy part of the blood, we have the right treatment to begin with. We want to rid the blood of the rubbish, and then begin to build—knows of nothing better than a mild course of arsenic. The water from springs that contain a trace of arsenic are invaluable in these cases. Doses of from 1–100 to 1–50th of a grain of arsenic are good. Mild topical applications of carbolic acid also may be employed. This treatment is fatal to the foreign organic matter in the blood, and the ulcers soon begin to grow less. Then we are ready to build. Without this constitutional treatment, the local treatment, is too frequently only time misspent.

At the same meeting Dr. Taft spoke as follows in regard to "gold foil of the heavier numbers:"

My practice lately has been to make the body of the filling with No 4 or 6, and to finish with the heavier foils, 30 and 60. A good deal depends upon the condition of the gold. If we could get the gold always just right the heavier foils, would then perhaps be better than the light for the whole. The advantage of using heavier foils on the surface is that a better finish is secursed. He finishes by laying on the flat, smooth pieces, and welding perfectly about the edges, by letting the heavy gold overlap the thin wall, or rather to hug down on the friable edge and thus secure and protect it. This can be better done with No. 60 in his hands than any other gold. We can save more of the form of the tooth with this method and No. than with thin foils."

Dr. Morgan on the "the treatment of alveolar abscesses remarked that he "thinks it unadvisable to attempt the cure of abscesses in the molars of a patient of strumous diathesis or in scrofulous patients. Treats through the pulp canal or alveolus, and frequently makes an opening to the point to be treated. Sometimes there appears to be a calcarious deposit on the point of a root, but analysis has found it composed entirely of animal matter. Sometimes simply cutting this away, or scraping the point of the root, or cutting away a little of the cementum and dentine at this point, is all the treatment necessary. Uses creosote in full strength frequently."

